# Summer surface temperature and socioeconomic data of Dutch residential zones, 2014

**DOI:** 10.1016/j.dib.2021.107255

**Published:** 2021-07-03

**Authors:** Bardia Mashhoodi

**Affiliations:** Landscape Architecture and Spatial Planning Group, Department of Environmental Sciences, Wageningen University & Research, P.O. Box 47, 6700 AA Wageningen, the Netherlands

**Keywords:** Land surface temperature, Summer surface temperature, Urban heat island, Environmental justice, Climate justice, Socioeconomic geography, Climate adaptation

## Abstract

The dataset combines and aggregates two data types at the scale of 2400 residential zones (“wijken”, in the terminology used by the Dutch Central Bureau for Statistics) of the Netherlands, 2014. The first type of data is summer surface temperature, the average of 40 dates in the summer of 2014, comprising the observations of four satellite images of four local overpassing times: MODIS Terra day (10:30 a.m.), MODIS Terra night (10:30 p.m.), MODIS Aqua day (1:30 p.m.), and MODIS Aqua night (1:30 a.m.). Second, ten variables describing the socioeconomic status of the residential zones: Western immigrants (%), Non-Western immigrants (%), Rental dwelling (%), Building age (median), Population age 65 or older (%), Population age 15-24 (%), Population age 14 or younger (%), Income per capita (x 1000 €), Property value (x 1000 €), Female minus male (%).

## Specifications Table

**Table:** 

Subject	Geography
Specific subject area	Summer land surface temperature and the socioeconomic characteristics of households in the residential zones of the Netherlands in 2014
Type of data	georeferenced datasets in the format of GIS polygon, GIS raster and SPSS with geographic reference.
How data were acquired	The raw data is acquired from MODIS satellite imagery and the Statistical Bureau of the Netherlands. The data, subsequently, is analysed by use of Arc GIS Pro software.
Data format	Raw Analysed
Parameters for data collection	Collection of satellite imagery data was according to three criteria: (1) provision of as many as possible satellite images from the summer of 2014, with roughly equal time intervals; (2) collection of as many as possible rasters cells which are not covered by cloud; (3) including as many as possible images.
Description of data collection	The data is collected from public, open data sources and subsequently analysed.
Data source location	Institution: Landscape Architecture and Spatial Planning Group, Department of Environmental Sciences, Wageningen University & Research, the Netherlands: https://www.wur.nl/en/Research-Results/Chair-groups/Environmental-Sciences/Landscape-Architecture-and-Spatial-Planning-1.htm Institution providing primary data and links: Statistics Netherlands (CBS): https://www.cbs.nl/ National Aeronautics and Space Administration (NASA): https://earthdata.nasa.gov/ ESRI (Environmental Systems Research Institute) Netherlands: https://www.esri.nl/nl-nl/home Latitude and longitude (and GPS coordinates, if possible) for collected samples/data: The Netherlands (52°22′N 4°53′E)
Data accessibility	With the article
Related research article	Mashhoodi, B., 2021. Environmental justice and surface temperature: Income, ethnic, gender, and age inequalities. Sustainable Cities and Society, 68, p.102810. https://doi.org/10.1016/j.scs.2021.102810[1].

## Value of the Data

• The dataset brings NASA's MODIS satellite imagery and socioeconomic data of the residential zones of the Netherlands together.
• Researchers in environmental science, human geography, urban planning, and health studies can benefit from the data.
• The data can be further used for studies on environmental justice, climate adaptation and the impact of climate change on energy consumption.

## 1 Data Description

**Fig. 1:**
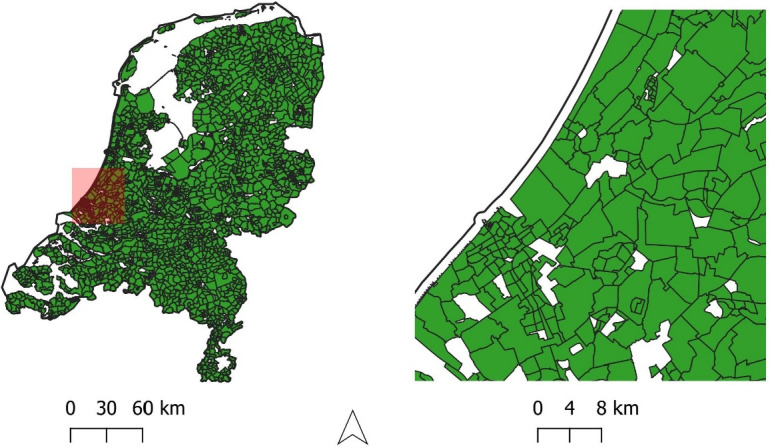
Case study areas: The residential zones of the Netherlands -the so-called Wijken (left), and a zoom-in preview of the zones (left).

To study environmental justice of land surface temperature, this article provides six types of data aggregated at the scale residential zones (Fig. 1): (1) land surface temperature during summer; (2) economic status of residents; (3) ethnic composition of residents; (4) presence of different age groups; (5) gender composition; (6) housing tenure and value. To do so, the following datasets are used, processed, and provided.

1. LST_Summer_Rasters_2014.gdb: geodatabase of the raw MODIS satellite imagery in the format of GIS raster. The name coding is as follows: *MOD_Day* refers to MODIS Terra day (10:30 a.m.); *MOD_Night* refers to MODIS Terra night (10:30 p.m.); *MYD_Day* refers to MODIS Aqua day (1:30 p.m.); *MYD_Night* refers to MODIS Aqua night (1:30 a.m.). The numbers in the rasters' name refer to the starting day of the eight days the images represent. For instance, *MOD_Day_153* is MODIS Terra day (10:30 a.m.), representing the average land surface temperature values of the days number 153 to 161 in 2014 [2].
2. Wijk_2014: the statistical bureau of the Netherlands' published data over the residential zones (Wijk) in 2014, in GIS polygon shapefile format.
3. Buildings_DEM: the raw data over the buildings' height in the format of 25 m × 25 m GIS raster (given the sheer size of the original file in polygons, the raster version is stored).
4. Buildings_Age: the raw data over the buildings' age in the format of 25 m × 25 m GIS raster (given the sheer size of the original file in polygons, the raster version is stored).
5. Aggregated_data: SPSS file with summer surface temperature and socioeconomic data of the residential zones of the Netherlands. Using the zones code (WK_Code), the data can be joined to the GIS file titled Wijk_2014.

### 1.1 Raw data

#### 1.1.1 MODIS satellite imagery

**Fig. 2:**
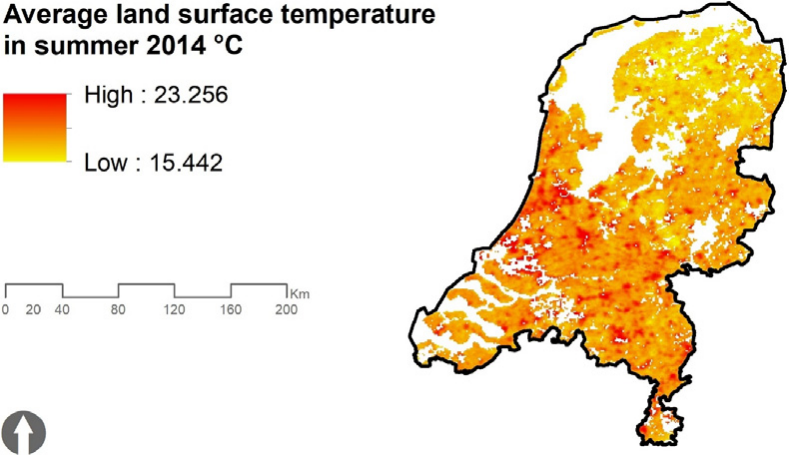
Average land surface temperature in the Netherlands, summer 2014.

The raw data of summer surface temperature consists of twenty MODIS satellite images representing five time periods and four overpassing local times, taken in June, July or August 2014 [2]. Each image is a raster file with a 1 km × 1 km resolution, representing the average value of eight days and nights at a particular overpassing local time. The images are taken from four different sources, each of which taken at a different local overpassing time: MODIS Terra day (10:30 a.m.), MODIS Terra night (10:30 p.m.), MODIS Aqua day (1:30 p.m.), and MODIS Aqua night (1:30 a.m.). Table 1 shows the time period of the images. See the files in the geodataset titled LST_Summer_Rasters_2014.gdb. The missing pixels in the surface temperature raster file, e.g. those covered by clouds in one of the twenty raw raster files, are not included in calculating the aggregated values (see Table 1 and Fig. 2)

**Table 1:** Time-periods of the satellite images.

Time-period
**T01**: 02 June 2014 - 09 June 2014
**T02**: 26 June 2014 - 03 July 2014
**T03**: 20 July 2014 - 27 July 2014
**T04**: 05 August 2014 - 12 August 2014
**T05**: 29 August 2014 - 05 September 2014

#### 1.1.2 Buildings

The so-called 3D BAG is a dataset of buildings shape, location, height, and construction year in the Netherlands [3]. Given the raw data's sheer size in polygon format, The raw data over the buildings' height and age in the 25 m × 25 m GIS raster format is attached to this manuscript (see the folders Building_DEM and Building_Age).

#### 1.1.3 Socioeconomic data

The source for socioeconomic data is the GIS shapefile published by the statistical bureau of the Netherlands containing social, economic, and accessibility data on the residential zones of the Netherlands, the so-called Wijken, in 2014 [4] (See the attached data in the folder Wijk_2014).

### 1.2 Aggregated dataset

Ultimately, the different data sources are aggregated at the residential zone scale (see the file Aggregated_data.sav). Using the zones code (WK_Code), the data can be joined to the GIS file titled Wijk_2014. Below, the data in the Aggregated_data file are presented.

#### 1.2.1 summer surface temperature

Satellite images are aggregated at the scale of the Netherlands' residential zones (wijken) – spatial boundaries defined by the statistical bureau of the Netherlands [4], and the average summer surface temperature of each zone is retrieved. To aggregate the stellite imagery rasters (1 km × 1 km) at the sale of residential zones, first, the rasters resampled at a finer scale (50 m × 50 m). Subsequently, the fine-scale raster file is aggregated at the scale of residential zones.

#### 1.2.2 Socioeconomic data

The dataset consists of ten variables describing the socioeconomic context of the residential zones in 2014:

• Western immigrants (%) – the percentage of residents with at least one parents from a European country, North America, Japan or Indonesia;
• Non-Western immigrants (%) – the percentage of residents with at least one parents from none of those mentioned above;
• Rental dwelling (%) – the percentage of dwellings in which the occupant is not the owner;
• Property value - the average value of properties in the zone;
• Building age - the median age of buildings with residential function, weighted by size;
• Population age 65 or older (%) – the percentage of residents aged more than 65 years;
• Population age 15-24 (%) - the percentage of residents aged between 15 and 24 years;
• Population age 14 or younger (%) - the percentage of residents younger than 14 years;
• Income per capita (x 1000 €) – the average annual disposable income per capita;
• Female minus male (%) – the percentage of female residents minus that of males.

The building age source is the Netherlands' buildings dataset, the so-called 3D BAG [3]. (See the raster file in folder Building_age and Building_DEM). The source of the other data is the statistical bureau of the Netherlands [4]. (see the file in folder Wijk_2014.)

## 2 Experimental Design, Materials and Methods

The dataset presented here brings two data sources together: the NASA satellite imagery and the Dutch comprehensive socioeconomic dataset of the residential zone. The five dates for satellite imagery were chosen based on three criteria: as many as non-polluted cells, roughly similar time intervals, and inclusion of as many as possible images. To aggregate the satellite images at the scale of residential zones, the raster file is resampled, as some of the rasters were located on the borders of two or more zones. Each raster is divided into 400 smaller rasters (50 m × 50 m), and the fine-grained raster file is used for spatial aggregation. Ultimately, the current dataset offer opportunities for experimental, explorative studies on environmental justice, climate adaptation and energy transition. Given the abundance of clean pixels, i.e. not polluted with obstacles such as clouds, of satellite imagery in the summer of 2014, the combination of the two datasets provides reliable data for studying the environmental justice of exposure to summer surface temperature for different socioeconomic groups (similar to [5], [6], [7]). The data could be used for setting urban cooling strategies at the most crucial location (see [8]). The dataset can further be utilised for studying the impact of urban heat islands on energy consumption (similar to [9]) and energy poverty (similar to [10]). The data could be combined with spatial data at the regional scale using principal component analysis method to set the context for the impact of summer surface temperature on energy consumption (similar to [11]). Ultimately, the dataset can be used to analyse different spatial scales (similar to [12]) and fuel consumption types (similar to [13]).

## Ethics Statement

The raw data of this study is provided by open, public GIS data sources, in full compliance with ethical requirements for publication in the journal of Data in Brief.

## CRediT Author Statement

**Bardia Mashhoodi**: Conceptualization, Methodology, Software, Data curation, Writing- Original draft preparation, Visualization, Investigation, Validation, Reviewing and Editing.

## Declaration of Competing Interest

The authors declare that they have no known competing financial interests or personal relationships which have or could be perceived to have influenced the work reported in this article.

## Appendix Supplementary materials

Image, application 1
